# P2X_7_ receptor activity regulation: the role of CD44 proteoglycan GAG chains

**DOI:** 10.1038/cddis.2015.340

**Published:** 2015-11-26

**Authors:** G E D D Moura, S V Lucena, M A Lima, F D Nascimento, T F Gesteira, H B Nader, E J Paredes-Gamero, I L S Tersariol

**Affiliations:** 1 Departamento de Bioquímica, Universidade Federal de São Paulo, São Paulo, Brazil; 2 Grupo de Pesquisa em Biomateriais e Biotecnologia, Universidade Bandeirante de São Paulo, São Paulo, Brazil; 3 Division of Developmental Biology, Cincinnati Children's Hospital and Research, Cincinnati, OH, USA; 4 Centro Interdisciplinar de Investigação Bioquímica, Universidade de Mogi das Cruzes, São Paulo, Brazil

P2X_7_ receptors have received special attention in the literature for their involvement in several diseases characterized by inflammatory processes such as cancer, arthritis, neurodegenerative pathologies and chronic pains.^1^ The P2X_7_ receptor is an adenosine 5′-triphosphate (ATP)-gated non-selective cation channel and its activation mediates the depolarization of an inward current due to a major influx of Na^+^ and Ca^2+^ into the cytosol, while a concomitant efflux of K^+^ is generated.^2^ The binding of ATP to the P2X_7_ receptors is tightly regulated by allosteric mechanisms that act either on their extracellular or on their intracellular/transmembrane domains.^3^ In our recently published manuscript in *Cell Death Discovery*,^4^ it was proposed that the cell surface glycosaminoglycan (GAG) chains from CD44 proteoglycans have a relevant functional impact on P2X_7_-receptor physiology, emerging as a new cofactor that is necessary for the full receptor activity and a new post-translational regulatory mechanism for purinergic signaling at the cellular level.^4^

We have shown that GAGs from the cell surface bind to the P2X_7_ receptor and facilitate the binding of ATP to the ligand-gated cation channel. The presence of GAGs at CHO cell surface greatly increases sensitivity to low concentrations of ATP and changes the main P2X_7_ kinetic parameters EC50, Hill coefficient and *E*max. Yet, in the absence of ATP, even the highest heparin concentration tested did not elicit a noticeable P2X_7_ activation. Furthermore, the allosteric block of P2X_7_ receptor current by extracellular Mg^2+^ ion were mitigated when GAGs are present. Our data argue for an allosteric sensitization of the receptor by GAGs. In addition, the formation, recruitment and the P2X_7_ pore dilation augmented in the presence of GAGs as demonstrated by the acceleration of cellular uptake of large molecules such as propidium iodide (MW 668 Da) and by the molecular dynamic simulations. Increase in *E*max of Ca^2+^ influx and acceleration of propidium iodide influx confirmed the potentiating effect of GAGs on native P2X_7_ receptors. Consequently, wild-type CHO-K1 cells were more sensitive to cell death induced by P2X_7_ agonists than its mutant CHO-745, defective in GAG biosynthesis.^4^

The ability of P2X_7_ to respond to a wide range of ATP concentrations reflects ATP binding to its three binding sites on the trimeric receptor of negative cooperativity, where partial ATP occupancy results in the opening of an intrinsic non-selective pore for small mono- and divalent cations, including Ca^2+^. On the other hand, full occupancy at high ATP concentrations triggers the pore dilation. Thus, the rate and extent of P2X_7_ sensitization determine the outcome of the receptor activation.^5^ We identified cell surface GAGs as key regulators of P2X_7_ receptor sensitization and pore dilation.^4^ Our data support a model in which GAG binding might overcome the conformational hindrances under conditions of partial agonist occupancy and thereby promote the long opening–gating mode (Figure 1).

Using confocal microscopy experiments, we have shown that P2X_7_ receptors co-localize with CD44 proteoglycans on the wild-type CHO-K1 cell surface, but not in the CHO-745 cell line. Moreover, we used immunoprecipitation followed by immunoblotting approaches to provide a direct biochemical evidence for physical association between soluble CD44 ectodomain (sCD44) and P2X_7_ receptor. CD44 proteoglycans are involved in a wide spectrum of physiological functions such as cell–cell and cell–matrix interactions, morphogenesis, cell migration, cellular differentiation and tumorigenic process.^6^ Also, it is important to mention that CD44 proteolytic cleavage products, sCD44 and CD44-ICD, serve as tumorigenic factors by enhancing cell proliferation/migration.^7^ Interestingly, ATP-mediated cytoplasmic Ca^2+^ influx by P2X_7_ receptors results in the CD44 ectodomain proteolytic shedding forming sCD44 products^8^ via P2X_7_ receptor stimulation of disintegrin and metalloproteinase-10 (ADAM10)-dependent proteolytic activity.^9^

CD44 proteoglycans and P2X_7_ receptors are involved in oncogenic processes and most malignant tumors do overexpress them. Tumorigenic cells overexpressing P2X_7_ receptors show enhanced engraftment ability and *in vivo* growth rate, enhanced invasiveness, increased expression of proliferation markers, reduced apoptosis and angiogenesis.^10^ Seemingly, CD44 overexpression is essential for the anchorage-independent growth, tumor growth and tumor-initiating ability of highly tumorigenic mammary epithelial cells.^11^ Our study suggests GAG chains from sCD44 as a hitherto physiological positive allosteric modulator of P2X_7_ receptor, where sCD44 is part of a regulatory positive feedback loop linking P2X_7_ receptor activation, which allows the intracellular response mediated by ATP cell signaling (Figure 1).

Ultimately, our study has several implications: (1) the presence of GAGs on the cell surface controls the rate of P2X_7_ sensitization and determines the outcome of receptor activation, whereas the absence of the GAGs at cell surface results in a hypo-functional P2X_7_ receptor with reduced agonist sensitivity; (2) the regulation of CD44 proteoglycans'/GAGs' fine structure may provide a mechanism for the cellular control of P2X_7_ activation; (3) an effective antagonist in an *in vitro* model may not serve for human therapeutic use if GAGs are used as the physiological positive allosteric modulator of P2X_7_ receptors, hence caution should be taken when investigating the pharmacological blockade of P2X_7_ in both *in vitro* and *in vivo* studies; (4) the activity of P2X_7_ receptors may be spatially and temporally coordinated with the CD44 proteoglycan expression in different cellular models and in various pathophysiological states (Figure 1).

## Figures and Tables

**Figure 1:**
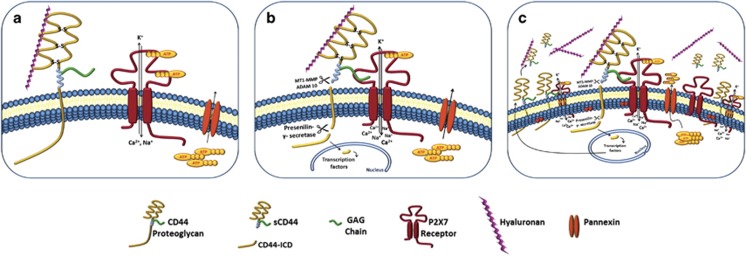
GAG chains from CD44 proteoglycan are a physiological positive allosteric modulator of P2X_7_ receptor. P2X_7_ stimulation on the surface of CHO cells is dependent on the extracellular concentration of ATP and P2X_7_ density, and expression of CD44 proteoglycan is dependent on their activation status. ATP-mediated P2X_7_ activation increases the cytoplasmic level of Ca^2+^, a universal second messenger. Ca^2+^ influx by P2X_7_ receptors results in the ectodomain proteolytic shedding of CD44 by activation of proteolytic enzymes, ADAM10 and presenilin-γ-secretase, forming as products sCD44 and CD44-ICD, respectively. CD44-ICD translocates to the nucleus and activates gene transcription. (**a**) The absence of the GAG chains from CD44 proteoglycan on the cell surface resulted in a hypo-functional P2X_7_ receptor with reduced agonist sensitivity. (**b**) GAG chains from CD44 are a positive allosteric modulator of P2X_7_ receptor; where sCD44 is part of a regulatory positive feedback loop linking P2X_7_ receptor activation and thereby allows the intracellular response mediated by ATP cell signaling. (**c**) The activity of P2X_7_ receptors may be spatially and temporally coordinated with the CD44 proteoglycan expression in different cellular models and in various pathophysiological states. CD44 proteoglycans and P2X_7_ receptors are involved in oncogenic processes; most malignant tumors overexpress P2X_7_ and CD44 receptors. CD44 proteolytic cleavage products, sCD44 and CD44-ICD, serve a tumorigenic process by enhancing the proliferation/migration of cells
